# C-Reactive Protein Triggers Cell Death in Ischemic Cells

**DOI:** 10.3389/fimmu.2021.630430

**Published:** 2021-02-10

**Authors:** Ahmed Sheriff, Stefan Kayser, Patrizia Brunner, Birgit Vogt

**Affiliations:** ^1^Pentracor GmbH, Hennigsdorf, Germany; ^2^Medizinische Klinik m.S. Gastroenterologie/Infektiologie/Rheumatologie, Charité Universitätsmedizin, Berlin, Germany; ^3^iAdsorb GmbH, Berlin, Germany

**Keywords:** CRP–C-reactive protein, ischemia/reperfusion injury, cardiovascular, COVID-19, inflammation

## Abstract

C-reactive protein (CRP) is the best-known acute phase protein. In humans, almost every type of inflammation is accompanied by an increase of CRP concentration. Until recently, the only known physiological function of CRP was the marking of cells to initiate their phagocytosis. This triggers the classical complement pathway up to C4, which helps to eliminate pathogens and dead cells. However, vital cells with reduced energy supply are also marked, which is useful in the case of a classical external wound because an important substrate for pathogens is disposed of, but is counterproductive at internal wounds (e.g., heart attack or stroke). This mechanism negatively affects clinical outcomes since it is established that CRP levels correlate with the prognosis of these indications. Here, we summarize what we can learn from a clinical study in which CRP was adsorbed from the bloodstream by CRP-apheresis. Recently, it was shown that CRP can have a direct effect on blood pressure in rabbits. This is interesting in regard to patients with high inflammation, as they often become tachycardic and need catecholamines. These two physiological effects of CRP apparently also occur in COVID-19. Parts of the lung become ischemic due to intra-alveolar edema and hemorrhage and in parallel CRP increases dramatically, hence it is assumed that CRP is also involved in this ischemic condition. It is meanwhile considered that most of the damage in COVID-19 is caused by the immune system. The high amounts of CRP could have an additional influence on blood pressure in severe COVID-19.

## Introduction

Inflammation in humans is deeply evolutionary rooted. A quick and intense inflammatory response is required for the efficient eradication of injury and was highly beneficial in times where external wounds or life-threatening infections where the main—if not only—cause of damage to the body (1). Inflammation as a weapon against environmental risks and triggers is unfortunately a two-edged sword, because it is dangerous when turned against the own body. In modern times, an elevated inflammatory function is thought to be associated with higher risk to develop atherosclerosis, diabetes and other age-related diseases, which are not caused by pathogens (2). But an enhanced immune system can not only facilitate cardiovascular disease, it also exacerbates acute incidents, which are “sterile.” In order to heal internal wounds, the body needs the inflammatory reaction to eliminate dead cells. At the same time activating proliferation and repair mechanisms as well as restoring tissue homeostasis is essential. This immune response is, however, since centuries specialized on the thorough eradication of cells around a wound to minimize the risk of infection. Even if these cells are only energy deprived but still viable. In the setting of an internal wound, as e.g., myocardial infarction or stroke, this effect is often destructive and threatening as it only aggravates deterioration and involves severe collateral damage (3, 4).

Likewise, an excessive immune reaction not justified by its external trigger induces more negative than positive effects on the body (5). During a systemic inflammatory response syndrome or sepsis the inflammation is so enormous and disproportionate that it causes widespread tissue injury and might finally result in multiple organ failure (6). Here, the whole body is often affected by inflammation showing hard to control hemodynamic instability.

Although a multitude of proteins are involved in inflammation, most of them do not actively participate in the elimination of pathogens or human cells (1). One of the acute-phase mediators directly involved in these pro-inflammatory processes is C-reactive protein (CRP) which was discovered by Tillett and Francis (7). CRP is well-established as one of the most reliable markers of inflammation, rising dramatically during any type of inflammation.

There are several very good and extensive reviews published, summarizing the role of CRP as unspecific inflammatory marker and its history from discovery to world-wide used lab marker (8–11). Even though CRP has been investigated in numerous clinical studies and its association and correlation with the progress of certain diseases is evidently clear (12–19), evidence that it is a mediator of the respective disease in humans was missing while strong evidence exists for different animal species (16, 20–22). In addition, CRP can dissociate into monomers physiologically, although it is still under debate if it thereby exerts different molecular functions than the pentameric form (23). The transition of pentameric CRP to monomeric CRP was described in specific inflammatory microenvironments (24, 25). Pro-inflammatory isoforms of pentameric and monomeric CRP were reported (26). The circulating CRP is pentameric and the actual source of all further processes. Despite this extensive in-depth knowledge, the widespread opinion is still that in humans CRP is only an unspecific biomarker.

We want to focus on the controversy/debate that CRP also in humans is not only a marker but an active pro-inflammatory protein, which contributes causally to the severity of tissue damage and the outcome of various diseases (27).

## CRP is an Active Inflammatory Protein

**Figure 1:**
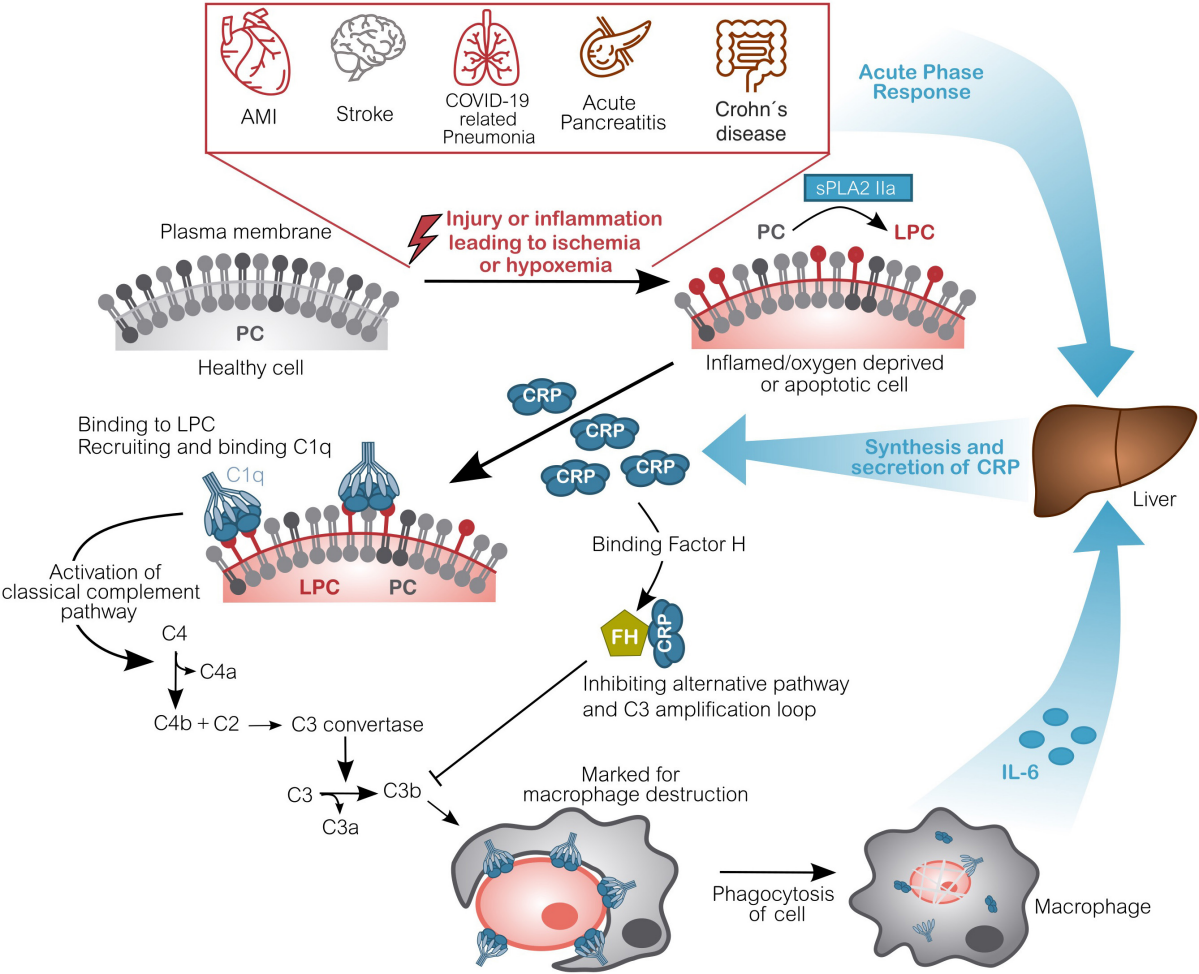
Molecular pathomechanism of CRP-mediated damage in ischemic or hypoxemic tissue. Inflammation or acute oxygen-deprivation happens for example in acute myocardial infarction (AMI), stroke, during COVID-19 related pneumonia, acute pancreatitis as well as during an acute Crohn's disease relapse. This leads to energy-depleted, hypoxic or even ischemic tissue. Cells within this tissue display a modified outer cell membrane: Phosphatidylcholine (PC) is converted into lyso-phosphatidylcholine (LPC) by phospholipase (sPLA2 IIa). Due to the lack of energy, this alteration cannot be reversed. CRP subsequently binds to LPC on anaerobic cells and recruits complement factors (C1q-C4), activating the classical complement pathway. These opsonized cells will be disposed by phagocytes, which in turn induce CRP synthesis by secretion of IL-6. CRP also binds Factor H, which inhibits the alternative complement pathway and actually protects healthy host cells from disposal (16, 22, 79). Although CRP is drawn pentameric, it should be noted that after binding to ischemic cells, the recruitment of C1q and particularly FH is potentially carried out by dissociated, monomeric CRP. Figure adapted from (41). AMI, Acute Myocardial Infarction; CRP, C-reactive protein; C1q, Complement component 1q; C2-C4(a/b), Complement component 2-4(a/b); FH, Factor H; IL-6, Interleukin 6; LPC, Lysophosphatidylcholine; PC, Phosphatidylcholine; sPLA2 IIa secretory phospholipase A2 type IIa.

CRP is secreted by the liver into the blood circulation where it efficiently detects and opsonizes bacteria upon their infiltration (28, 29). By marking these pathogens, it initiates their phagocytosis via activation of complement. This mechanism is mainly caused by the binding of CRP to the phosphorylcholine groups in the membrane of bacteria. These groups, however, are also present in all human cells, albeit not accessible on healthy cells. Cells that are apoptotic, necrotic, energy-depleted or simply exposed to inflammatory environments, often being acidic and hypoxic, undergo conformational and biochemical changes in their membrane (30). One of these being the partial hydrolyzation of phosphatidylcholine (PC) to lyso-phosphatdiylcholine (LPC) by another acute-phase-protein, namely the secretory phospholipase A2 type IIa (sPLA2 IIa) (31–34). This makes the phosphorylcholine group accessible to the binding of CRP. Hence, CRP irreversibly marks dying, dead, damaged or hypoxic/ischemic cells. Subsequently, the classical complement pathway is activated and the CRP-marked cells are disposed by phagocytosis (35–40). See Figure 1 for the hypothesized pathomechanism of CRP after an acute phase response caused by inflamed or hypoxic/ischemic tissue.

In the setting of an internal wound this generates a vicious cycle: The primary inflammation triggered by e.g., ischemia (e.g., acute myocardial infarction or stroke) activates a switch to anaerobic metabolism and a striking synthesis and secretion of CRP mediated by IL-6. CRP is circulated to the wound, where it mediates the disposal of dead and dying cells. High CRP concentrations cause more cells to be marked, including still viable cells, which could have regenerated their membrane after restoration of the oxygen flow and switching back to aerobic metabolism. The phagocytosis of these cells in turn produces IL-6, inducing the synthesis of additional CRP, subsequently amplifying the immune response. Thereby, CRP causally contributes to the tissue damage and scarring after an incident [Figure 1, adapted from (41)] (22, 42–44). It should be noted that CRP is synthesized and secreted as a pentamer by the liver, however, it can dissociate into monomers within the microenvironment of the inflamed/ischemic tissue and might exert the drawn functions as monomer (Figure 1).

Although not every step of this molecular mechanism has been proven and shown in detail, there have been convincing proof-of-concept studies in animals substantiating this hypothesis. A large body of data obtained either in rats, porcine models or *in vitro* in the infarcted myocardium of humans has demonstrated that CRP plays an active role in exacerbating ischemia and reperfusion-induced damage (16, 22, 45–50).

Recent studies exceedingly revealed that CRP modulates signaling cascades besides the classical complement pathway (51–54). This shows that CRP has direct physiological effects on not only inflammation but also the function of e.g., endothelial cells, be it their metabolism, differentiation or migration (55–57). In the context of endothelial cells, it is also discussed if CRP might have protective effects in atherosclerotic lesions. It was shown that, although CRP induces complement activation, it protects the bound cells from the formation of final complement components (58, 59), mainly by recruiting complement factor H (60). The role of CRP in atherosclerosis is however still under debate and cannot be compared to acute ischemic incidents regarding the circulating concentration of CRP.

## CRP has an Effect on Blood Pressure

One of these recent studies showed a direct, quick and extreme effect of CRP on blood pressure in rabbits (61). Human CRP was intravenously injected *in vivo* to reach a level of 50 mg/L. It dramatically reduced the arterial blood pressure within minutes, while the heart rate remained the same and did not counteract as expected and necessary to maintain the oxygen supply of the organism. The effect persisted for more than 17 min.

While it has been shown that blood pressure and heart rate as well as adrenergic receptor (AR) signaling can affect CRP concentrations (62–64), a direct influence of CRP on hemodynamic variables has hardly been investigated so far. Other *in vivo* studies administering CRP were performed in rats and humans (65–67). However, the recombinant CRP used in the studies never achieved concentrations of 50 mg/l and blood pressure was measured not directly but only hours after injection (65, 67). Hence, the acute effects might have been overlooked.

The direct and acute effect of high CRP concentrations on blood pressure gives a first hint, why critically ill patients, suffering from e.g., sepsis or acute pancreatitis, can develop hardly controllable hemodynamic variables with preceding elevated CRP levels (68).

After seeing such a dramatic drop in blood pressure the influence of CRP on AR signal transduction was investigated *in vitro* (61). ARs signal via calcium (Ca^2+^) as second messenger was measured in real-time. CRP triggered calcium signaling in a dose-dependent manner in two different human cell lines, expressing either α- or β-adrenergic receptors. Further, CRP induced an additional calcium increase that came on top of AR agonists phenlyephrine or isoprenaline. This indicates a molecular mechanism that is independent of adrenoceptor activation.

Effects of CRP on endothelial cells have mostly been attributed to its effect on endothelial nitric oxide synthase (eNOS), although studies have to be interpreted with care, as contaminating products in commercial CRP solutions, such as sodium azide, were possibly often causally involved (69–71). eNOS is activated by an increase in intracellular calcium, leading to vasodilatation (72, 73), which could explain the drop of blood pressure *in vivo*. The mechanism underlying CRP's induction of calcium influx is still undiscovered and should be investigated in future studies.

Therefore, it was demonstrated that one molecule of the inflammation cascade has an influence on blood pressure. The direct influence of other inflammatory mediators needs to be investigated.

## What We Can Learn From Studies Using CRP as a Therapeutic Target Molecule

Many reports following the hypothesis that CRP has pathological effects suffer from a mean to shut down its activity. Knock-out mice do not represent appropriate models, because CRP in mice fulfills different functions than in humans and does not act as an acute-phase protein (74). Other animal models like e.g., transgenic rabbits expressing human CRP have been used but here most of the investigations focused on atherosclerosis and not on acute incidents (75).

In humans the use of CRP-lowering drugs has so far not been successful, since CRP as acute-phase protein increases drastically within hours and its circulating levels need to be lowered quickly in an acute setting. This cannot be achieved by targeting its synthesis or using approaches, which take several days to affect CRP levels (16, 21, 76).

Selective immuno-adsorption of CRP from the serum avoids these problems and has been shown to efficiently reduce CRP concentrations by ~60% within hours (77–79). The elimination of pathogenic substances from the blood by means of extracorporeal apheresis is an established therapy in the clinical routine of numerous diseases.

CRP apheresis aims to remove CRP from the blood plasma after an infarction to reduce acute tissue damage and ischemic reperfusion damage (41). It has most extensively been utilized after acute myocardial infarction (AMI).

### CRP Depletion After Myocardial Infarction

Patients recovering from a heart attack often suffer from a reduced quality of life and a very high risk of subsequent serious complications (e.g., heart failure, arrhythmias, second heart attack, death), which imposes an enormous burden on the healthcare system. It was observed that this risk correlates significantly with the size of myocardial injury and scarring (80, 81).

It has long been known that inflammation, mainly mediated by the innate immune system, expands myocardial injury. However, anti-inflammatory strategies to mitigate myocardial necrosis have so far failed, perhaps because these processes are also necessary for the healing and repair of the heart (3, 4, 82, 83). Whereas, baseline CRP values are recognized as a determinant of the incidence of cardiovascular disease (12, 14), serum CRP concentration after AMI correlates with the clinical outcome (19, 42, 44, 84–87). It is textbook knowledge that high CRP peaks in the first 72 h after AMI correlate with larger infarct size and higher mortality and the incidence of additional adverse cardiovascular events (42–44). This has been documented for four decades and is consistent with the described pathological function of CRP to eliminate cells in the area at risk (27, 30, 88, 89). This region comprises cells that might recover after revascularization and reperfusion, but are eventually destroyed by immune-mediated mechanisms, as explained above and shown in detail in numerous experimental approaches specifically focusing on AMI (20, 46, 48, 85, 90, 91).

It has therefore been suggested earlier to target CRP in AMI, but the therapeutic approaches were not clinically relevant or sufficiently rapid (16, 92–94). This changed when it was shown that specific extracorporeal removal of CRP by CRP apheresis resulted in a significant reduction of the infarct area and stabilization of the left ventricular ejection fraction (LVEF) in a preclinical trial in pigs (22, 50). An interesting observation is that the scar morphology of the animals after CRP apheresis was completely different from controls, supporting the hypothesis that CRP is directly involved in tissue destruction and scarring after the incident (22). Consequently, CRP apheresis has been used in one clinical trial and two case reports in patients with ST-elevation myocardial infarction (STEMI) (77, 79, 95).

In the CAMI-1 trial it was investigated whether specific depletion of CRP can reduce the size of myocardial infarction in humans. Eighty-three patients were included and the treatment was safe and well-tolerated (79). The extent of increase of CRP concentration during the first 32 h after STEMI significantly correlated with the infarct size in control patients. Patients with similar initial CRP increase who subsequently underwent CRP apheresis, had smaller infarct sizes and better LVEF and wall motion (strains) when compared to control patients. Surprisingly, some patients treated with CRP apheresis had not even minor infarct scars and a normal LVEF (96).

It is possible that the supply bottleneck in ischemic tissue does not immediately lead to tissue necrosis, but rather to a conversion of the energy metabolism into anaerobic glycolysis, which leads to a significant lack of energy of the individual cardiomyocytes (97). Afterwards, the cardiomyocytes go into stunning until the metabolism switches back to the aerobic one which eliminates the energy deficiency. This suggests that the cardiomyocytes only survive if they are not marked by CRP and thus disposed of by phagocytes.

### CRP in Stroke

This effect can possibly be applied to other ischemia-mediated injuries as for example stroke. Here, similar mechanisms to AMI take place and inflammation plays a crucial role during the occlusion but also subsequent therapeutic reperfusion of the tissue (98, 99). The inflammatory response after stroke has been discovered to be a key prognostic factor for patients (100, 101). High CRP concentrations during the first 48 h after the incident significantly predict immediate and long-term mortality as well as the overall prognosis (13, 101, 101–103). This is supported by pre-clinical evidence in a stroke model in rats and subsequent application of high amounts of human CRP (49). This strongly supports the hypothesis that CRP plays a similar pathological role as in AMI.

## CRP May Increase Destruction of Tissue in COVID-19

SARS-CoV-2 can lead to COVID-19 and induces pulmonary fibrosis and cardiac complications in a minor percentage of infected individuals, among other organ deterioration (104). A major therapeutic approach focuses on the treatment of acute respiratory distress syndrome, as it is the leading cause of mortality, followed by cardiac and septic complications. In the severe course of the disease a massive increase in the CRP concentration accompanied by an initial cytokine storm is followed by pulmonary fibrosis (105, 106). Intra-alveolar edema and hemorrhage is a common observation in the lungs of COVID-19 patients which leads to ischemic alveolar tissue. It may be assumed that CRP itself triggers tissue damage by binding to these ischemic cells and is thus also causally involved in the enlargement of the destroyed tissue and contributes to irreversible tissue destruction (22, 79). Both IL-6 and CRP increase dramatically in the course of clinical manifestation of COVID-19 (107) and rising CRP levels were shown to significantly predict the respiratory decline in patients (105). CRP levels also correlate with CT findings of COVID-19 patients (108). These findings further support the hypothesis that a significant increase in CRP is a signal of lung deterioration and disease progression.

Complement deposits were found by pathologists in the lungs of deceased COVID-19 patients. Among them was especially C1Q. Since C1Q also inhibits antiviral CD8+ effector T-cell responses, a higher frequency of CD8+PD1+ T-cells was found, possibly indicating T-cell exhaustion (109). Despite the depletion of the T cells, massive destruction in the lungs is found along with the extreme levels of CRP in the aggravated COVID-19 patients. C1Q is known to bind CRP after CRP binds the lysophosphatidylcholine of ischemic cells (22).

In addition, cardiac involvement was observed by MRI analysis in 78% of patients, and persistent myocardial inflammation was observed in 60% of patients with recent COVID-19 disease, regardless of pre-existing conditions, the severity and overall course of the acute disease and the time from initial diagnosis (110). Myocardial inflammation was suggested as the underlying mechanism (111, 112).

CRP apheresis provides a therapeutic approach to rapidly decrease the high CRP levels in COVID-19 patients before lung deterioration can progress (113). Until now, the therapeutic option of reducing the extremely high amount of CRP has been used once in the early phase of COVID-19 and in end-stage patients with one case being reported (113, 114). A clinical study in this indication would be beneficial and is currently planned.

## Conclusion and Outlook

The understanding of CRP has undergone two basic transitions. First, CRP has been established as a general biomarker of inflammation and infection in clinical practice. Then, its role as a stable and highly useful prognostic factor for cardiovascular and cerebral disease in healthy individuals has been widely acknowledged and utilized (17, 115). However, the characterization of CRP as not only a biomarker but also a mediator or even trigger of destruction of tissue in humans is still widely ignored (27, 46, 49).

CRP as an archaic protein of the innate immune system physiologically disposes cells and responds to almost every change in tissue homeostasis. From the perspective of the body's energy balance, one has to ask oneself why CRP is produced in large quantities by the liver in situations where it seems wiser to keep a proper energy reserve. Certainly not to provide us with a meaningful biomarker. It is more logical to recognize that it is provided in potentially septic wounds during enemy defense, to eliminate further cells, so that they do not serve the enemy for propagation. This function has unfortunately a negative effect on typically aseptic inner wounds. Further, the demonstration of a direct effect of CRP on blood pressure shows us that the molecular functions of CRP are still not comprehensively described and that the role of this protein is largely underestimated in critically ill patients.

Accepting CRP as an active inflammatory protein offers the promising possibility to therapeutically target CRP whenever the inflammatory reaction is too extensive or not beneficial. Ongoing and future clinical trials will illuminate whether this therapeutic approach will continue to prove its value.

## Author Contributions

AS and PB conceptualized and wrote the first draft. SK and BV made substantial corrections.

## Conflict of Interest

AS was CEO and shareholder of Pentracor GmbH. SK was employed by Pentracor GmbH. BV was shareholder and employee of Pentracor GmbH. PB was employed by iAdsorb GmbH.
